# Why Are Some Snakes More Terrifying and What Is Behind the Fear?

**DOI:** 10.3390/ani15050731

**Published:** 2025-03-04

**Authors:** Daniel Frynta, Markéta Janovcová, Hassan Sh Abdirahman Elmi, Iveta Štolhoferová, Veronika Rudolfová, Kateřina Rexová, David Sommer, David Král, Daniel Alex Berti, Eva Landová, Petra Frýdlová

**Affiliations:** 1 Department of Zoology, Faculty of Science, Charles University, 128 00 Prague, Czech Republic; daniel.frynta@natur.cuni.cz (D.F.); markii47@seznam.cz (M.J.); rabiic23@amoud.edu.so (H.S.A.E.); iveta.stolhoferova@natur.cuni.cz (I.Š.); veronika.rudolfova@natur.cuni.cz (V.R.); kloskacka@centrum.cz (K.R.); david.sommer@natur.cuni.cz (D.S.); david.kral@natur.cuni.cz (D.K.); daniel.berti@volny.cz (D.A.B.); eva.landova@natur.cuni.cz (E.L.); 2 Department of Biology, Faculty of Education, Amoud University, Borama 25263, Somaliland

**Keywords:** fear, evolutionary psychology, cross-cultural comparison, Viperidae, Elapidae, Boidae, ophidiophobia

## Abstract

Snakes are stimuli inducing an ancestral fear response in humans and other primates. Certain snakes evoke more subjective fear than others. True vipers are high-fear-eliciting snakes for both African and European respondents. This can be explained by the evolutionary experience of human ancestors in Africa. The question arises as to how snakes living in the Americas and Australia, with which humans have no evolutionary experience, will be evaluated. While these snakes belong to broader taxonomic groups that have distant relatives in the Old World, they have evolved independently for tens of millions of years. We prepared a set of 32 pictures depicting eight American pit vipers, eight Australian elapids, eight constrictors, and eight colubrids and asked the respondents to rank these stimuli according to the fear these snakes evoke. Here, we show a high cross-cultural agreement between evaluations by African and European respondents. Snakes characterized by a robust body shape, such as American pit vipers, Australian death adders, pythons, and boas, were the most fear-evoking. The body width was the strongest predictor of evoked fear. The contribution of coloration and pattern of the stimulus to the fear response was not proved. This supports the view that the patterns of fear are not dependent on direct experience, but its underlying mechanisms are shared cross-culturally.

## Simple Summary

People are deeply afraid of snakes, and this fear is believed to have evolved in our ancient ancestors, primarily in Africa. The most dangerous venomous snakes are vipers and elapids, with previous studies showing that people are particularly fearful of vipers. We wanted to explore how people would react to snakes from the Americas and Australia—regions humans settled only recently. We created a set of 32 snake images and asked participants from Africa and Europe to rank them based on the fear they evoked. Surprisingly, both groups ranked the snakes similarly, despite their vastly different experiences with snakes. The most fear-inducing snakes were American pit vipers (e.g., rattlesnake and bushmaster), which are distant relatives of Old-World true vipers. Australian death adders strongly resembling true vipers also ranked highly, as did some large constrictors, such as American boas and Australian pythons. Interestingly, Australia’s highly venomous snakes ranked lower, alongside harmless American colubrids. The rankings were best explained by the snake’s body shape—people were more afraid of thick-bodied snakes. This suggests that humans have inherited simple rules for judging the danger of unfamiliar snakes based on their appearance.

## 1. Introduction

### 1.1. Snakes as Ancestral and Still Relevant Fear-Eliciting Stimuli for Humans

Snakes are highly salient stimuli that evoke fear [1,2,3] and, in some cases, phobias in humans [4]. Since the innate fear of snakes is also well-documented in many other primates, it is hypothesized that this fear arose due to selective pressure from snakes, initially through predation by large constrictors such as modern pythons or boas [4,5,6]. Evidence from contemporary populations of hunter-gatherers in tropical regions demonstrates that attacks by large constrictors remain common even today [7]. The emergence of modern venomous snakes marked a shift in selective pressures, with mortality caused by their defensive bites becoming a significant factor [8,9]. Globally, approximately 125,000 people die annually from snakebites [10,11,12], particularly in Asia [13] and Africa. In Africa alone, the annual burden is estimated at 268,471 cases of envenomation, 12,290 deaths, and 14,766 amputations [14,15]. In comparison with Africa, the number of deaths reported from Central Europe is negligible [16,17,18].

Nevertheless, not all snakes are venomous. Humans may respond to this fact in two distinct ways, which are not mutually exclusive. One strategy is to fear all snakes indiscriminately, merely based on recognizing the stimulus as a snake. This strategy is supported by treating all limbless reptiles as a distinct category [19] and experiments showing that all examined snake species are rated among the most fear-inducing animals [20]. The conceptual background explaining this strategy is provided by studies reporting that the primate brain is equipped with early detection of snake stimuli processed by a koniocellular visual pathway arising from the retina and connecting to the lateral geniculate nucleus, the superior colliculus, and the pulvinar. This shortcut directly informs subcortical centers like the amygdala and enables immediate response to snakes [5,21,22,23,24,25]. There are studies suggesting that the early response involves the detection of a curvilinear shape of the snake body [26]. Nevertheless, subsequent studies revealed that the curvilinear shape itself is not sufficient [27,28], and the attention of researchers was redirected to the detection of snake scales [28,29,30,31,32].

The second strategy involves assessing the potential danger of a snake based on its external appearance and adjusting the fear response accordingly. For example, subjective fear and psychophysiological responses are higher for viperids when compared to fossorial snakes, with the latter tending to elicit disgust rather than fear [33,34,35]. Studies comparing multiple and diverse snake stimuli consistently reported clear and systematic differences in the levels of fear they elicit [6,36,37]. This may be attributed to perceptual biases and/or cognitive processes evaluating the external appearance of the stimulus. The presence of features predicting danger, similarity to a prototypic fear-evoking snake, and categorization may be involved. Let us assume that not only the fear of snakes itself but also the cognitive processes involved in assessing the danger of specific snake stimuli predominantly arose during the long evolutionary history of interactions with snakes.

### 1.2. Are Vipers Prototypical Fear-Evoking Snakes?

A recent study compared subjective fear elicited by 48 snake stimuli from four groups of Old-World snakes differing in morphotype and danger level: vipers, elapids, sand boas, and colubrids. The first two groups are venomous, whereas the sand boas are non-venomous, and colubrids are either non-venomous or only mildly venomous. Respondents from both Africa (Somalis) and Europe (Czechs) consistently rated vipers as the most fear-inducing group. Surprisingly, cobras and mambas, which are at least as dangerous to humans, induced less fear than vipers not only in European but also in African respondents exposed to the presence of these elapids in their everyday lives [6]. Thus, we do not immediately recognize these neurotoxin-equipped snakes as dangerous unless they actively display threat signals when disturbed [37]. Similarly, vipers were perceived as the most fear-inducing stimuli in a cross-cultural study utilizing Azerbaijani and Czech respondents, surpassed by only one species of cobra in a threatening posture [36]. These findings provide the basis for a hypothesis that vipers are likely a prototypical stimulus for perceiving snake danger, given their long evolutionary interaction with human ancestors in Africa [6]. However, it could be argued that boid snakes in the above-mentioned study [6] were represented by sand boas—small burrowing animals that do not resemble the typical larger snakes of the family Boidae as modern Malagasy and American boas. Moreover, large pythons, representing another group of constrictors dangerous for primates, were not included. Therefore, it cannot be reliably ruled out that giant constrictors could induce fear comparable to that of vipers. However, this assumption has yet to be verified.

### 1.3. Human Evolution and Interactions with Snakes in the Past

The evolution of human primate ancestors occurred exclusively in the Old World, initially in Eurasia and later primarily in Africa, where modern venomous snakes existed as early as the Oligocene, i.e., over 20 million years ago [6]. The earliest representatives of the genus *Homo* were known from East Africa around 2 million years ago (mya). *H. ergaster* soon expanded back into Eurasia (~1.8 mya, [38,39]). However, modern humans (*H. sapiens sapiens*) evolved again in Africa approximately 300,000 years ago [40,41]. Around 60,000 years ago, they migrated to Asia [42,43], interbreeding with local populations such as Neanderthals and Denisovans [44,45], and gradually spread to other continents, first to Australia (~30,000 years ago) and later to the Americas (~15,000 years ago). It follows that all human populations have had a prolonged evolutionary history with African snakes. In sharp contrast to African snakes, American and Australian snakes were encountered by humans just recently and until the advent of modern European colonization exclusively by Indigenous populations, such as Aboriginal Australians and Native Americans.

### 1.4. Why Study Human Response to American and Australian Snake Species?

Populations in Africa and Eurasia typically have direct experience only with snakes native to their regions, while encounters with snakes from other continents are limited to tourism, zoological gardens, or media. More importantly, they lack any evolutionary experience with American and Australian snakes. These snakes thus provide ideal stimuli for testing how Old-World respondents evaluate unfamiliar snakes. Assessments of American and Australian snakes are likely to be based solely on the superficial characteristics of the stimuli, avoiding any contamination by respondents’ direct and cultural experience. It is especially advantageous when searching for general traits that make snake species scarier. We can benefit from the natural diversity of external snake appearance, enabling us to utilize naturally occurring stimuli rather than artificially creating them. Even after tens of millions of years of separate evolution, distinct snake lineages retain certain external characteristics of the phylogenetic groups from which originally diverged. This allows us to investigate whether people still evaluate these American or Australian snakes like their Old-World distant relatives. Additionally, once the focus is on cross-cultural agreement driven by innate perceptual and cognitive mechanisms, it is crucial to use stimuli with which respondents have no direct experience.

### 1.5. Evolutionary History and Composition of Snake Faunas in the Americas and Australia

The snake fauna of the Americas and Australia is diverse; therefore, we will focus on the groups of snakes that are relevant to the study of human fear and examine their evolutionary history.

The most prominent venomous snakes in the Americas are pit vipers (subfamily Crotalinae) of the broadly delimited viperids (family Viperidae). The split between the true vipers of Africa and Eurasia (subfamily Viperinae) and the common ancestor of the primitive vipers (Azemiopinae) and pit vipers (Crotalinae) is deep (about 50 mya, [46]). Pit vipers (Crotalinae) originally diversified in Asia, where they rapidly increased their speciation rate (in the Oligocene about 30 mya) and invaded North America through the Bering Land around the Oligocene/Miocene boundary [46,47,48]. The American pit vipers constituted a monophyletic group [46,49,50] which initially continued in rapid speciation in the American continent [46,47] and underwent further diversification of body size, morphology, and ecological strategies [51]. Certain lineages of the American pit vipers, such as rattlesnakes of the genus *Crotalus*, bushmasters of the genus *Lachesis*, and neotropical lanceheads (*Bothrops*), reach large sizes and are lethally venomous for humans [52].

In addition to pit vipers, another branch of highly venomous snakes has been present in the Americas for a long time. These are coral snakes that split from their Asian elapid ancestors at the beginning of the Miocene (approximately 22 mya, [53,54,55]). American elapids are generally small in size and often exhibit warning coloration—aposematism [56,57]. As such coloration may affect the fear evaluation (e.g., [58,59], but see [60,61]) and its function in snakes is a matter of current discussion (e.g., [57,62]), we omitted coral snakes in this study.

The snake fauna of the Central and South Americas is also characterized by the presence of an ancient lineage of boid constrictors (subfamily Boinae), including some larger or even gigantic forms of boid snakes, the largest of which is the green anaconda (*Eunectes murinus*). The homeland of the Boinae is probably the South American continent, as they have been present in the fossil records of Brazil since the Paleocene [63,64,65], although their closest fossil relatives are known from North America and Europe [66,67,68]. There are fossil records suggesting that boids were also present in Central America [68,69] and even in Florida [70] in the Early Miocene, well before the closure of the Panama Isthmus and the great American faunal interchange. These species are non-venomous, but some may be dangerous to humans owing to their large size.

The Australian snake fauna is highly specific, it is dominated by the venomous elapids related to Sea Kraits of the genus *Laticauda* [50,55]. All elapid snakes of Australia are currently assigned to the subfamily Hydrophiinae. This name was originally used exclusively for Sea Snakes which are now considered to be just a single and highly derived lineage of a monophyletic group that diversified in the terrestrial environment. The ancestor of Australian elapids (Hydrophiinae) appeared in Australia during the Miocene epoch [54]. Among venomous snakes, they were the first to colonize Australia and neighboring New Guinea, where they gradually diversified in terms of morphology [71,72] and venom composition [73,74]. Among the Australian elapids, there are multiple exceptionally venomous genera, such as black snakes (*Pseudonaja*), brown snakes (*Pseudechis*), taipans (*Oxyuranus*), tiger snakes (*Notechis*), and death adders (genus *Acantophis*). Some of them are even considered the most lethal snakes to humans worldwide [75,76,77,78]. Moreover, specialized morphotypes of elapids emerged in the Australia de novo, resembling outward appearance snakes from other continents to which they are not closely related. In the context of this stimuli, the death adders bearing a striking resemblance to Old World vipers are the most important.

In addition to venomous elapids, there are also non-venomous pythons in Australia. These snakes are constrictors some of which reach considerable size. The earliest python fossils date back to the early Tertiary period (Eocene) and originated from Europe [79]. It is assumed that they later dispersed to other continents [80,81]. The earliest fossil records of pythons from Australia are not precisely dated but originate from the period between the late Oligocene and the middle Miocene [82,83].

### 1.6. The Aims and Hypotheses

This study aimed to investigate the fear evoked by snakes from the Americas and Australia in African and European respondents—a fear of snakes they have had no direct contact or evolutionary experience. In our previous studies, participants evaluated species they were familiar with either directly (still present in their environment) or at least evolutionarily (due to shared evolutionary history). We detected a high cross-cultural correspondence with Somali, Azerbaijani, and Czech respondents all rating true vipers as the most fear-inducing snakes [6,36]. This high concordance between respondents, despite their exposure to different snake faunas and risks of envenomation, supported the hypothesis that fear responses to snakes evolved during the shared evolutionary history of these populations. The specific, shared evolutionary history between human ancestors and viperids may be behind this cross-cultural consensus on recognizing the vipers as both dangerous and clearly morphologically distinct. The current study addresses this issue by examining fear evoked by unfamiliar snake species. We focused on highly venomous snakes and large constrictors, while colubrids were included as a control. Investigating the fear of those snake species that have coexisted with humans for a very short period (taken from an evolutionary perspective) will help to conclude whether humans a priori recognize certain types of dangerous snakes even if they have never encountered the snake species themselves, culturally or evolutionarily. Specifically, we addressed the following questions:

1. Do American and Australian snakes that elicit the greatest fear resemble true vipers (subfamily Viperinae) in appearance? If so, this would support the hypothesis that vipers are prototypical stimuli.
2. Do Australian elapids, similarly to Old-World elapids, evoke relatively low levels of fear compared to that elicited by some other snakes, in particular vipers?
3. How does fear elicited by large constrictors (e.g., boas and pythons) compare to other snake groups?
4. Which visceral characteristics of snake stimuli are the best predictors of elicited fear?
5. How strong is the cross-cultural agreement in ranking snake stimuli based on elicited fear?

## 2. Materials and Methods

### 2.1. Selection of Snake Species and Preparation of the Stimuli

In this study, we examined fear evoked by snakes belonging to four distinct phylogenetic categories, each represented by eight picture stimuli (=species):

1. Pit vipers of the American clade of the subfamily Crotalinae. A recent study delimited five phylogenetic groups of this clade [51], two of which (Lachesis and the North American group) are sister lineages whose common ancestor branched first from the ancestor of the remaining ones. We included at least one species of each group, while the most speciose group was represented by four species. The Lachesis clade was represented by the South American Bushmaster (*Lachesis muta*); the North American clade by the Neotropical Rattlesnake (*Crotalus durissus*); the Bothriechis clade by the Highland Eyelash Pit viper (*Bothriechis schlegelii*); the Porthidium clade by the Rainforest Hognosed Pit viper (*Porthidium nasutum*); and finally the Bothrops clade by the Amazonian Toad-headed Pit viper (*Bothrocophias hyoprora*) and three species of the genus *Bothrops*: Urutu Lancehead (*Bothrops alternatus*), jararaca (*B. jararaca*) and Mato Grosso Lancehead (*B. mattogrossensis*). All these species are equipped with solenoglyphous fangs and are distributed in Central and/or South America.
2. Boas (Boidae) and pythons (Pythonidae). We included six species of Central and South American boas of the subfamily Boinae: An Argentine boa (*Boa occidentalis*), the Garden Tree Boa (*Corallus hortulana*), two anacondas (green—*Eunectes murinus* and yellow—*E. notaeus*), and two Rainbow boas (Argentine—*Epicrates alvaresi* and Western—*E. cenchria*). These species represent all principal clades of New World boids corresponding to genera, except the Antillean *Chilabothrus* [84,85]. We also added two species of Australian pythons, the Southwestern Carpet Python (*Morelia imbricata*) and the Rough-scaled Python (*M. carinata*). All these species are non-venomous constrictors. Only giant species such as anacondas may represent a certain danger for humans.
3. Australian elapids of the subfamily Hydrophiinae. The first offshoot of this clade is represented by the Yellow-faced whipsnake (*Demansia psammophis*). The next split formed two speciose clades, the first one further splits into a branch represented in our stimuli by death adders (*Acanthophis antarcticus* and *A. praelongus*) and (*Pseudechis australis*), and the branch represented by the taipan (*Oxyuranus scutellatus*), the Ringed brown snake (*Pseudonaja modesta*), and Eastern brown snake (*P. textilis*). The Curl snake (*Suta suta*) represents the second-most speciose clade in our stimuli.
4. Central and South American colubrids (Colubridae). This group was aimed as a control to venomous groups. Two subfamilies of colubrids are equally represented in our set of stimuli; four species belong to the subfamily Colubrinae (Clark’s forest racer—*Dendrophidion clarkii*, Giant Parrot Snake—*Leptophis ahaetulla*, Salmon-bellied racer—*Mastigodryas melanolomus*, Brown Vinesnake—*Oxybelis aeneus*), while the others to the subfamily Dipsadinae (Lehmann’s ground snake—*Atractus lehmanni*, Velvety Swamp Snake—*Erythrolamprus typhlus*, Brown-banded watersnake—*Helicops angulatus*, and Lema’s ground snake—*Lygophis dilepis*). Dipsadinae are basal offshoots of Colubridae, which is sometimes elevated to a family level [50,86].

**Figure 1:**
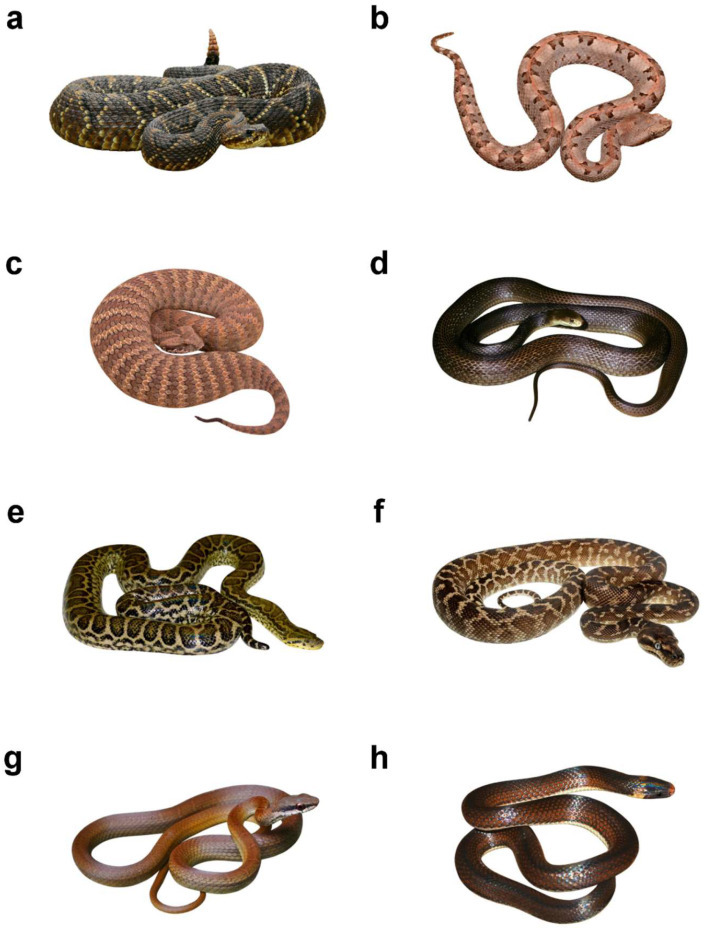
Examples of visual stimuli. (**a**)—South American Rattlesnake (*Crotalus durissus*), photo by Leandro Avelar (CC BY-SA 4.0); (**b**)—Rainforest Hognosed Pit viper (*Porthidium nasutum*), photo by Andrew DuBois (CC BY-NC 2.0); (**c**)—Common Death Adder (*Acantophis antarcticus*), photo by Graham Armstrong (CC BY-NC 4.0); (**d**)—Taipan (*Oxyuranus scutellatus*), photo by John Wombey (CC BY 3.0); (**e**)—Yellow anaconda (*Eunectes notaeus*), photo by Olga Šimková and Markéta Janovcová; (**f**)—Rough-scaled python (*Morelia carinata*), photo by Scott Eipper (CC BY-NC 2.0); (**g**)—Salmon-bellied racer (*Mastigodryas melanolomus*), photo by Josue Ramos Galdamez (CC BY-NC 4.0); (**h**)—Lehmann’s ground snake (*Atractus lehmanni*), photo by Josh Vandermeulen (CC BY-NC-ND 4.0).

For each snake species on the list, we selected an appropriate photograph from the authors’ archives and online databases (Supplementary Table S1). To minimize potential bias from background and stimulus size, the images were digitally edited to place the snakes on a white background and standardized to a similar size. The final stimuli were printed as 100 × 150 mm photographs. An example of experimental stimuli is provided in Figure 1.

### 2.2. African and European Respondents

The African part of the study was conducted on the campus of Amoud University in Borama, Somaliland. Participants were primarily undergraduate students from various disciplines who voluntarily took part in the experiment. The respondents originated from the Awdal region (the region where the University is located), other provinces of Somaliland, and neighboring Somali-speaking countries. A total of 216 Somali respondents completed the task (see Supplementary Table S2), comprising 193 men and 23 women, with a mean age of 21.82 years (range 17–35).

The European respondents were recruited from Czech students. Although testing was conducted at universities in Prague, participants originated from various regions of the Czech Republic, including smaller towns and villages. The sample consisted of 167 respondents (31 men and 136 women), with a mean age of 20.59 years (range 17–30).

Prior to their participation, we asked the respondents to read and sign the written consent to participation in the experiment and the processing of the personal data. The data were further processed anonymously.

### 2.3. The Task

The task was personally administered by the authors (see contributions). We provided instructions to small groups of respondents, in Africa primarily in Somali (H.A.E.), and/or in the other two official languages of Somaliland (Arabic—D.F., D.A.B., and English). Then, each respondent performed the task individually on a separate table. The tables were situated in a well-lit room of the university cafeteria, which we booked for the forenoon period. At the start of the task, a respondent stood in front of a table and was provided with a set of 32 pictures packed in a random order. Participants were instructed to imagine the pictures as representations of real animals. They were then asked to place all stimuli on the table in a random arrangement. Assistance was occasionally required to ensure that the stimuli were oriented correctly, with the top margins facing the top of the table. The task involved selecting the picture of the most fear-evoking animal first, followed by the second most fear-evoking, and so on, until the least fear-evoking stimulus was selected. At the end of the task, respondents held the entire set of pictures ordered by their fear ratings. Participants were also asked to provide their age, and their gender was recorded. The task took approximately 15 min for most respondents. The final picture order was coded as ranks, ranging from 1 (most fear-evoking) to 32 (least fear-evoking).

### 2.4. Extraction of Morphological and Coloration Characteristics

The first step to assess which components of the external appearance of the snake stimuli contribute to evoked fear was a selection of main traits characterizing the shape, pattern, and coloration of each snake picture. To describe the body shape of the snake stimuli, we extracted nine morphological characteristics from the stimulus pictures. We employed Image Tool [87] to measure total body length, body width (at mid-body), head length, head width, neck width, tail width (close to cloaca), and eye diameter (all traits are in millimeters). We also measured the perimeter and body area of the snake silhouettes, both in pixels. For this purpose, we ran the Image J software (https://imagej.net/ij/) [88]. Both these programs are used in studies of human emotions elicited by visual stimuli [89].

To characterize the coloration and pattern of the stimuli, we extracted the pixel values of each photograph in the hue–lightness–saturation (HSL) color space using the software Barvocuc 2.0 [90] and following the method used in previous studies [89,91], formerly described in [92]. This software was specifically developed for analyses of animal pictures. The color angle definitions we adjusted according to human perception of depicted snakes as follows: red (332°; 16°), orange/brown (16°; 39°), yellow (39°; 80°), green (80°; 180°), blue (180°; 275°), purple (275°; 314°) and pink (314°; 332°). The color value extracted from the photograph represents the number of pixels of each color in the photograph. In addition to seven chromatic colors, we counted black (lightness < 18%), gray (saturation < 10%), and white (lightness > 80%) pixels. We also calculated the values of lightness and saturation and their standard deviations (SD of these characteristics refers to the overall complexity of the lightness/saturation pattern). Moreover, the software (Barvocuc) applied a mathematical function of the Sobel operator and extracted the value that may be viewed as another measure of picture complexity [93,94]. The values of traits characterizing the shape, pattern, and coloration of examined snake stimuli are provided in Supplementary Table S3.

### 2.5. Data Analysis

Given that the data consisted of ranks, we utilized non-parametric statistical methods, which are well-suited for analyzing such datasets. To quantify the level of agreement among respondents, Kendall’s coefficient of concordance (*W_t_*) was computed using the irr package [95]. This coefficient is suitable for our type of data [96]. To evaluate differences in the mean ranks of individual stimuli, we initially applied the Friedman test to confirm the significant effect of species. Subsequently, we applied the post hoc Friedman-Neményi test for reliable multiple comparisons among the individual stimuli, producing a matrix of *p*-values. These analyses were conducted using the PMCMRplus package version 1.9.12 [97].

Additionally, Redundancy Analysis (RDA), implemented in the vegan package version 2.6-6.1 [98], was employed to assess the proportion of variance in the dataset explained by factors such as country, gender, age, and their interactions. The vegan package is the most common software for RDA [99]. Means and median values of ranks were also calculated for each stimulus or species. To derive more intuitive scores that increase with fear (as opposed to decreasing, like the original ranks and their means) and scale from 0 to 100, we applied the following transformation:

Fear = 100 − (100 × (median rank − 1)/(the number of examined stimuli − 1)).

These transformed values were further analyzed.

To examine associations between fear scores and morphological or coloration characteristics of the stimuli, we calculated non-parametric Spearman correlation (r_s_). Comparisons of fear elicited by different snake categories were conducted using the Kruskal–Wallis test followed by Neményi post hoc comparisons, implemented in the PMCMRplus package. Cross-cultural agreement in fear scores between African and European datasets was quantified using Spearman rank correlation.

All calculations were carried out within the R environment (https://www.r-project.org/) [100]. For the cluster analyses, we employed Statistica 9.1 [101], which enabled us to identify groups of stimuli treated similarly by respondents. A dissimilarity matrix (1—Pearson’s *r*) was extracted from the ranking dataset, and Ward’s clustering method was applied. This software is regularly employed in similar studies [6,20,102].

## 3. Results

### 3.1. Agreement Among Respondents and Multivariate Patterns

We found significant agreement among 216 African respondents (Somalis) as well as among the 167 European ones (Czechs). Kendall‘s coefficients of concordance (*W_t_*) were 0.311 (χ^2^ _(31)_ = 2086, *p* << 0.0001) and 0.282 (χ^2^ _(31)_ = 1461, *p* << 0.0001), respectively. The descriptive statistics for each stimulus are given in Supplementary Table S4.

The RDA with permutation test revealed that the effects of gender (*p* = 0.18) and age (*p* = 0.13) on the evaluation of the stimuli are negligible. The best model includes the country (Somaliland vs. Czech Republic) as the only factor constraining 3.86% of the variation in the entire data set (ANOVA: F_(1,381)_ = 15.32, *p* < 0.001). Thus, we continued to analyze the African and European datasets separately.

### 3.2. Patterns of Elicited Fear and Post Hoc Comparisons Among Individual Snake Stimuli

Friedman tests proved that the effect of the stimulus on perceived fear was highly significant in both Somali and Czech datasets (*p* << 0.0001). Thus, we calculated Friedman–Neményi comparisons among stimuli. Out of 496 post hoc comparisons among the stimuli, 337 (67.9%) and 299 (60.3%) were significant in the Somali and the Czech datasets, respectively (*p* < 0.05, for the matrices of *p*-values, see Supplementary Tables S5 and S6).

The neotropical rattlesnake (*Crotalus durissus*, Figure 1a) was rated as the most fear-inducing stimulus by both African and European respondents, significantly ahead of most other stimuli. Of the following ten most fear-eliciting species, seven species appeared in the list by African as well as European participants (*Acanthophis antarcticus*, *Boa occidentalis*, *Bothrops alternatus*, *Bothrops jararaca*, *Eunectes murinus*, *Lachesis muta*, *Morelia carinata*). The following species ranked among the next top ten in Africa: second *Bothrops alternatus*, third *Lachesis muta* (Crotalinae), fourth *Helicops angulatus*, fifth *Eunectes murinus*, sixth *Boa occidentalis*, seventh *Acanthophis praelongus*, eighth *Bothrops jararaca*, ninth *Acanthophis antarcticus*, tenth *Morelia carinata*, and eleventh *Eunectes notaeus*. In Europe, the corresponding rankings were as follows: second *Lachesis muta*, third *Acanthophis antarcticus*, fourth *Morelia imbricata*, fifth *Bothrops alternatus*, sixth *Porthidium nasutum*, seventh *Bothrops jararaca*, eighth *Bothriechis schlegelii*, ninth *Morelia carinata*, tenth *Eunectes murinus*, and eleventh *Boa occidentalis*.

### 3.3. Comparing Fear Elicited by Main Phylogenetic Groups of Stimulus Snakes

**Figure 2:**
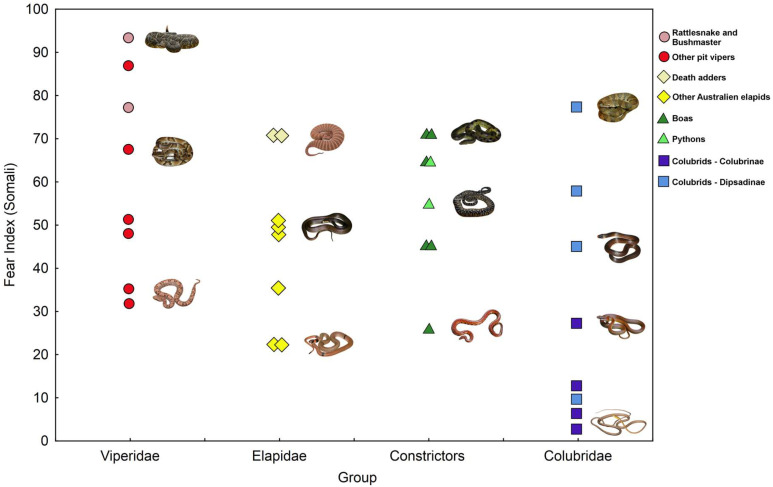
Plot of fear elicited by four phylogenetic categories of snakes in African (Somali) respondents.

**Figure 3:**
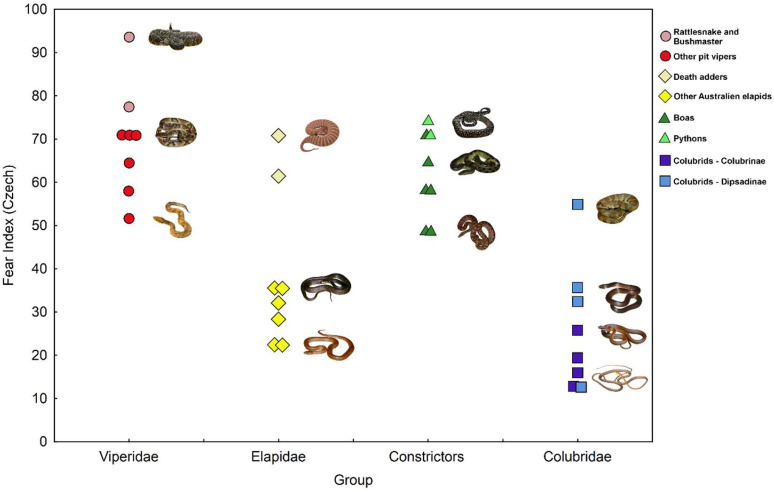
Plot of fear elicited by four phylogenetic categories of snakes in European (Czech) respondents.

In both Africa and Europe, the first third of positions were occupied with the only exception by the pit vipers (*Crotalus*, *Lachesis*, *Bothrops,* and *Bothriechis*), death adders (*Acantophis*), boas (*Boa* and *Eunectes*), and pythons (*Morelia*), while the opposite tail of rankings was occupied mainly by colubrids and the rest of elapids (Figure 2 and Figure 3). Among the seven stimuli ranked by African respondents as least fear eliciting, five were colubrids (32nd *Leptophis ahaetulla*, 31st *Oxybelis aeneus*, 30th *Lygophis dilepis*, 29th *Dendrophidion clarkia* and 26th *Mastigodryas melanolomus*—all except *Dendrophidion* representing the subfamily Dipsadinae), and two elapids (27th *Suta suta* and 28th *Pseudonaja modesta*). In the European data, all colubrids (except 17th *Helicops angulatus*) and elapids (except both *Acantophis*) were placed on 13 positions of snakes with the lowest fear values (Figure 3, Supplementary Table S4).

We also performed a formal test comparing fear elicited by four principal phylogenetic groups of snakes, the photographs of which we used as stimuli in this study. In both African and European data, the highest fear was elicited by the American pit vipers (mean values: 61.7 and 69.8, respectively), followed by pythons and boas (55.2 and 61.7), but Australian elapids (46.6 and 38.7) and colubrids (30.0 and 26.2) elicited less fear. Nevertheless, we failed to confirm the effect of the phylogenetic group on fear in African data (Kruskal–Wallis test: χ^2^ _(3)_ = 6.27, *p* = 0.0992), while in the European data, this effect was highly significant (χ^2^ _(3)_ = 18.83, *p* = 0.0003). This enabled us to perform post hoc comparisons between the snake groups. The fear index calculated from European data was significantly higher in pit vipers than in colubrids (*p* = 0.0007) and Australian elapids (*p* = 0.0396). Also, pythons and boas elicited significantly higher fear than the colubrids (*p* = 0.0098).

We performed a cluster analysis to identify groups of stimuli that received mutually correlated rankings. The main split revealed for the African dataset was between (1) the branch including Crotalinae (*Bothrops*, *Crotalus*, *Lachesis*, *Bothrops* except *B.matogrosensis*) + *Acantophis* + *Helicops* + *Boidae* (except *Corallus* and *E. cenchria*) and (2) the rest of examined stimuli (Supplementary Figure S1), while the European dataset resulted in three clusters containing (1) all Crotalinae + *Acantophis* + *Helicops*, (2) Boinae and (3) Hydrophiinae + Colubridae (Supplementary Figure S2).

### 3.4. Explaining Elicited Fear by Visceral Traits of the Snake Stimuli

**Figure 4:**
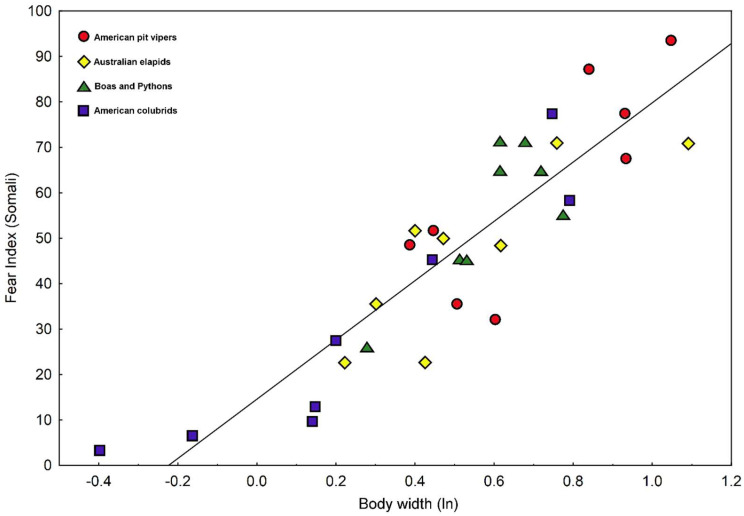
A plot of fear elicited by individual snake stimuli in the African (Somali) respondents against the natural logarithm of the body width of the stimulus.

**Figure 5:**
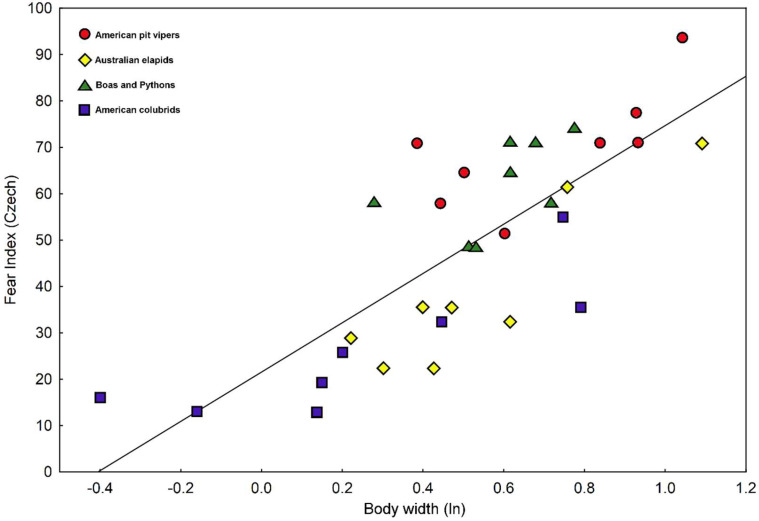
A plot of fear elicited by individual snake stimuli in the European (Czech) respondents against the natural logarithm of the body width of the stimulus.

We calculated correlation coefficients between fear values and 24 traits characterizing body shape, pattern, and coloration of the snake pictures (Supplementary Table S7). None of the 15 traits related to coloration and pattern was a significant predictor of the evoked fear. In contrast, multiple morphometric traits were clearly correlated with fear. Fear calculated from the African dataset was significantly associated with following six traits: body width (0.889), perimeter (−0.866), area (0.794), neck width (0.703), head width (0.684), and tail width (0.641), while fear assessed in European respondents with just four traits: body width (0.783), perimeter (−0.700), head width (0.672), and neck width (0.570). Thus, in both Africa and Europe, the best predictor of evoked fear was body width measures at mid-body (Figure 4 and Figure 5). The significant predictors of fear were, however, closely mutually correlated.

Multiple regression approach improved the correlations up to 0.957 for African (predictors in the model: body width, β = 0.345; tail width, β = 0.278; and perimeter β = −0.481) and 0.820 (head width, β = 0.868; and eye diameter, β = −0.489) for European datasets (Supplementary Table S8).

### 3.5. Cross-Cultural Agreement in Ranking Snakes According to Subjective Fear

**Figure 6:**
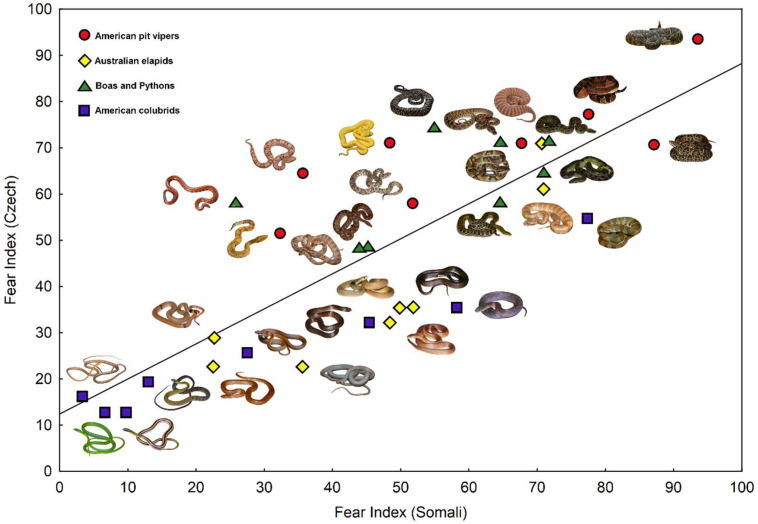
Plot of fear elicited by individual snake stimuli in the African (Somali) respondents against the corresponding values provided by European (Czech) ones. The Spearman correlation coefficient (*r*_s_) was equal to 0.805.

We plotted the relationship between fear of individual snake stimuli as evaluated by African and European respondents to visualize cross-cultural agreement (Figure 6). The correlation (*r*_s_) among the fear values extracted from the African and the European datasets was equal to 0.805 (*p* < 0.0001).

## 4. Discussion

### 4.1. Vipers or Constrictors?

The vast majority of highly venomous snakes worldwide belong to two distinctly different groups: vipers (Viperidae) and elapids (Elapidae). In our set of snake stimuli, vipers were represented by American pit vipers, while elapids were represented by the Australian subfamily Hydrophiinae. These two groups diverged from their Old-World relatives at least 20–25 million years ago but still retain certain ancestral characteristics. The question was whether these groups would elicit significantly stronger fear in respondents compared to our control group, which consisted of non-venomous American colubrids, as well as non-venomous American boas and Australian pythons.

In both the African and European datasets, the highest subjective fear was elicited by representatives of American pit vipers of the subfamily *Crotalinae*, such as rattlesnake (*Crotalus*), bushmaster (*Lachesis*), and neotropical lanceheads (*Bothrops*). This aligns with the hypothesis that the prototype of a fear-inducing snake stimulus is represented by vipers. However, among the most fear-inducing species were not only pit vipers and morphologically similar Australian death adders (*Acanthophis*) but also large constrictors, including American boas (*Boidae: Eunectes* and *Boa*) and Australian pythons (*Pythonidae: Morelia*). Thus, large-bodied constrictors also elicit significant fear, suggesting that vipers cannot be definitively confirmed as the exclusive group of snakes inducing high fear. Given that large-bodied constrictor snakes likely exerted selective pressure on primate populations earlier in evolutionary history, with vipers emerging as a threat only later [21,46,103,104,105], we cannot exclude the possibility that this fear response generalized to include both these groups of snakes. This interpretation assumes that vipers and constrictor snakes can be reliably distinguished. If this is not the case, it is possible that vipers are indeed the exclusive prototype of a fear-inducing snake, but large-bodied boas and pythons elicit high levels of fear just due to their superficial resemblance to vipers. An alternative explanation for why humans are the most afraid of both vipers and great constrictors is that they share a key morphological feature—a thick body (see Section 4.3).

The remaining two groups of snakes represented in the stimulus set elicited significantly less fear. These included other members of the Australian elapid lineage and, as expected, the predominantly non-venomous colubrids. This pattern, i.e., American pit vipers and constrictors (boas and pythons) evoking more fear than Australian elapids and American colubrids, was evident in ratings provided by both African and European respondents, although statistically significant differences among these four snake groups were found only in the European data. One possible explanation relates to the fact that the stimuli reflected natural diversity within taxa, and consequently, there were species that superficially resemble members of other groups. For instance, Australian elapids such as death adders (*Acanthophis*) were rated as highly fear-inducing by respondents, likely due to their strong resemblance to true vipers. Thus, the respondents were not able to fully distinguish snakes belonging to principal phylogenetic clades. On the contrary, they categorized them into certain morphotypes that only partly correspond to phylogenetic lineages, which reduced the “power” of phylogenetic lineages as an explanatory variable (see under Section 4.3).

### 4.2. A Paradox of Elapids: Extreme Toxicity but Low Fear Induction

The Australian elapids, except death adders, elicited relatively weaker subjective fear in humans compared to other snake groups, a surprising finding given that this lineage includes some of the most venomous snakes worldwide. A similar paradox has been observed in Africa, where mambas and cobras often ranked lower on the scale of the most fear-inducing local snakes [6].

In the Old World, however, one lineage of elapids developed a unique and highly effective behavior for deterring threats: the threat display known as *hooding*. This innovation, originally developed by the ancestors of cobras and mambas and further perfected in certain cobras, involves the expansion of a hood to signal danger [106]. Interestingly, a less sophisticated form of hooding evolved independently in certain Australian elapids, such as the tiger snake (*Notechis*, [107]).

When cobras are depicted in a hooding posture, they attract significantly more visual attention [108] and can ultimately elicit fear levels equal to or even exceeding those evoked by vipers [37]. The combined findings indicate that humans respond with full fear only when a cobra actively signals its presence. In this context, fear is a result of cobra behavior rather than an intrinsic human trait. Hooding evolved as a defensive mechanism to protect snakes from potential predators, including humans [37]. It has been speculated that an additional evolutionary step in response to threats from humans and other predators was the development of venom-spitting behavior in some cobras [106].

### 4.3. Predictors of Fear: Body Shape Versus Pattern and Colors?

The predictor of subjective fear is more likely to be body shape rather than patterns and colors, which can vary significantly among related species or even within conspecifics. We clearly confirmed the superiority of morphometric predictors over coloration and pattern. As innate categorization processes are assumed to rely on relatively simple cues, we expected that the average fear elicited by a stimulus would be largely explained by a single morphometric trait or a combination of a small number of such dimensions. In a previous study, which was carried out with the same populations of respondents but with different stimuli, this dimension was identified as midbody width [6]. In this study, body width appeared again as the best predictor of evoked fear in both the African and European datasets. Nevertheless, the small number of examined stimuli does not provide enough power to conclusively compare the effects of multiple morphometric traits, which are mutually highly inter-correlated.

The relationship between the size of a venomous snake and its danger to humans is not particularly strong, as small snakes can possess extraordinarily potent venom [75,109,110,111]. Moreover, fang morphology is also important [112]. However, larger snakes are capable of producing greater quantities of venom and often have longer fangs [113]. Consequently, large-bodied species, in general, are frequently ranked among the most dangerous, and body size serves as a useful predictor of risk. In our study, we found that among the examined morphological traits, snake mid-body width was the best predictor of evoked fear. It is reasonable to assume that this trait effectively predicts the snake’s weight and, therefore, its metabolic capacity, which contributes to venom production.

### 4.4. Cross-Cultural Agreement

If not only the fear of snakes itself but also the perceptual and cognitive processes involved in assessing the dangerousness of specific snake stimuli predominantly arose during the ancient evolutionary history of encounters with snakes, we would expect that fear-based evaluations of individual stimuli by respondents from different continents would be largely similar. Indeed, we repeatedly found such significant cross-cultural concordance.

In this study, the correlation between fear ratings of snake stimuli provided by African and European respondents was high (*r*_s_ = 0.805). This value aligns closely with correlation coefficients observed in previous cross-cultural studies employing similar methodologies: 0.826 [36], 0.738 [6], and 0.739 [37]. Cross-cultural agreement thus accounts for the majority of the average subjective fear elicited by individual stimuli. However, this does not imply that there are no differences between the evaluations of the same stimuli by Africans and Europeans. Multivariate analysis indicates that these differences contribute only a small fraction of the total variability in the rankings of the stimuli. The confirmation of a substantial cross-cultural concordance supports the hypothesis that the evaluations are predominantly governed by perceptual and cognitive constraints shared by African and European populations due to their shared evolutionary history. Interestingly, similarly high or even higher cross-cultural concordance has been previously observed in the evaluation of snake stimuli based on another criterion, beauty, as noted between Papuans and Europeans and among other populations from various continents [114,115]. In the case of snakes, but not other animal groups, beauty ratings tend to positively correlate with fear ratings of the same stimuli [19,36].

The levels of agreement among respondents from a single country in ranking snake stimuli by subjective fear were comparable to those reported in the aforementioned studies. There is no evidence suggesting that the evaluation of entirely unfamiliar snakes—likely ones with which the respondents have never had direct encounters—was notably more difficult for them.

## 5. Conclusions

In this study, African and European respondents evaluated snakes originating from the Americas and Australia—species with which they could not have had direct encounters or any evolutionary experience. Their assessments were thus expected to be free of biases towards specific species, relying solely on individual and/or evolutionary experience with snakes from their respective regions. High concordance between African and European respondents, despite their exposure to different snake faunas and risks of envenomation, supports the hypothesis that fear responses to snakes evolved during the shared evolutionary history of these populations. We showed that New World pit vipers and also boas and pythons induce the highest fear, while the opposite is true for American colubrids and Australian elapids, except the death adders resembling vipers. Further examination of the data showed that fear evoked by a stimulus snake can be successfully predicted by the body width of the snake, and this association is especially strong when fear evaluation provided by the African respondents is considered. We concluded that the patterns of evoked fear fairly resembled those previously reported in studies examining fear evoked by Old World snakes. Thus, the fear response is determined primarily by the body form of the snake stimulus and has little to do with coloration, pattern, and/or direct experience with a particular species.

## Data Availability

The original data associated with this manuscript are available in the Supplementary Materials.

## Supplementary Materials

The following supporting information can be downloaded at: https://www.mdpi.com/article/10.3390/ani15050731/s1, Figure S1: Results of clustering stimuli according to ranking provided by African (Somali) respondents; Figure S2: Results of clustering stimuli according to ranking provided by European (Czech) respondents; Table S1: Full list of the experimental stimuli; Table S2: Dataset for Somali and Czech respondents; Table S3: Extracted morphological and color characteristics; Table S4: Descriptive statistics for each stimulus; Table S5: Results of post hoc Friedman–Neményi test for Somali respondents; Table S6: Results of post hoc Friedman–Neményi test for Czech respondents; Table S7: Spearman coefficients of correlation for fear index and characteristics of the stimuli.
